# Supplementary material for: C-NHEJ without indels is robust and requires synergistic function of distinct XLF domains
Source: Nat Commun. 2018 Jun 27;9:2484. doi: 10.1038/s41467-018-04867-5 (PMC6021437; doi:10.1038/s41467-018-04867-5)
Supplement: Supplementary file 1 — Supplementary Information [file 41467_2018_4867_MOESM1_ESM.pdf]

C-NHEJ without indels is robust and requires synergistic function of distinct XLF domains

Ragini Bhargava, Manbir Sandhu, Sanychen Muk,

Gabriella Lee, Nagarajan Vaidehi, and Jeremy M. Stark

Supplementary Information

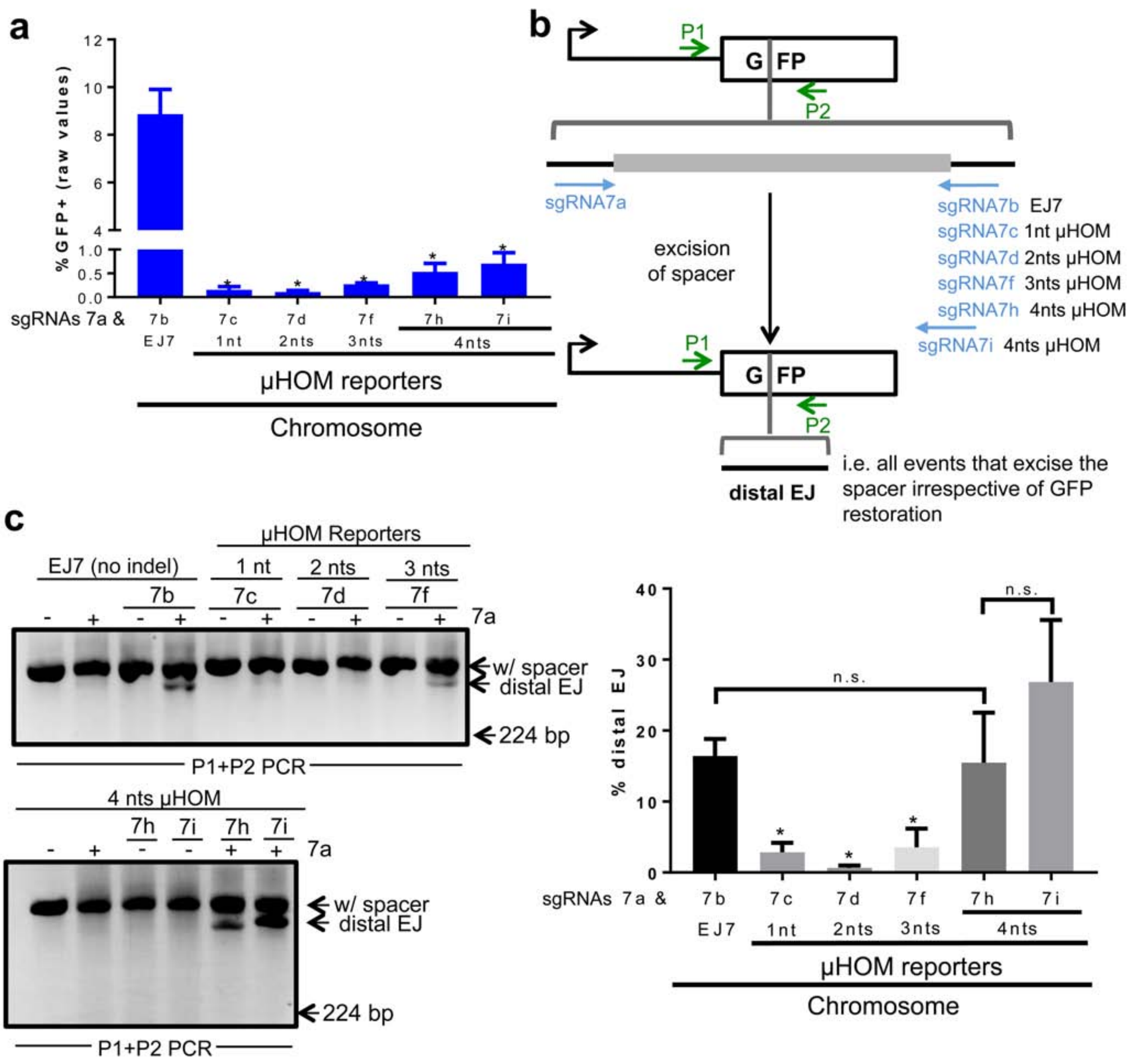

Supplementary Figure 1. **Evaluating the EJ7-GFP and the microhomology reporter assays for %GFP+ products, as well as total distal EJ, using a PCR-based assay. (a)** Shown is the frequency of GFP+ cells (raw values, not normalized to transfection efficiency) from each of the reporter assays from Fig. 1b and Fig. 3b in WT mESCs. N=6. Error bars represent s.d. *p<0.0001 microhomology events compared to the no indel repair event (EJ7-GFP) using an Unpaired t-test with the Holm-Sidak correction. **(b)** Schematic of a PCR-based assay to measure total distal EJ (% distal EJ). For each of the reporters in Fig. 3a, all distal EJ events between the two Cas9-induced DNA DSBs, regardless of GFP restoration, excises the spacer sequence, which can be measured by PCR with primers P1+P2 (sequences shown in the Methods). **(c)** Distal EJ analysis. Cells were transfected with sgRNA(s)/Cas9 as described in the Methods, scaled to 6 well, and including 200 ng pgk-puro plasmid. The day after transfection, cells were selected in 1.8 µg/ml puromycin for two days to enrich for transfected cells, prior to harvesting genomic DNA for amplification. Shown are representative agarose gel images of the PCR analysis showing the amplicon with the spacer region present (w/ spacer) or absent (distal EJ) for EJ7 and each of the microhomology reporters. Also shown is the % distal EJ for each of the reporters. N=3. Error bars represent s.d. *p<0.005 compared to EJ7 using an One-Way ANOVA with the Dunnett's test.

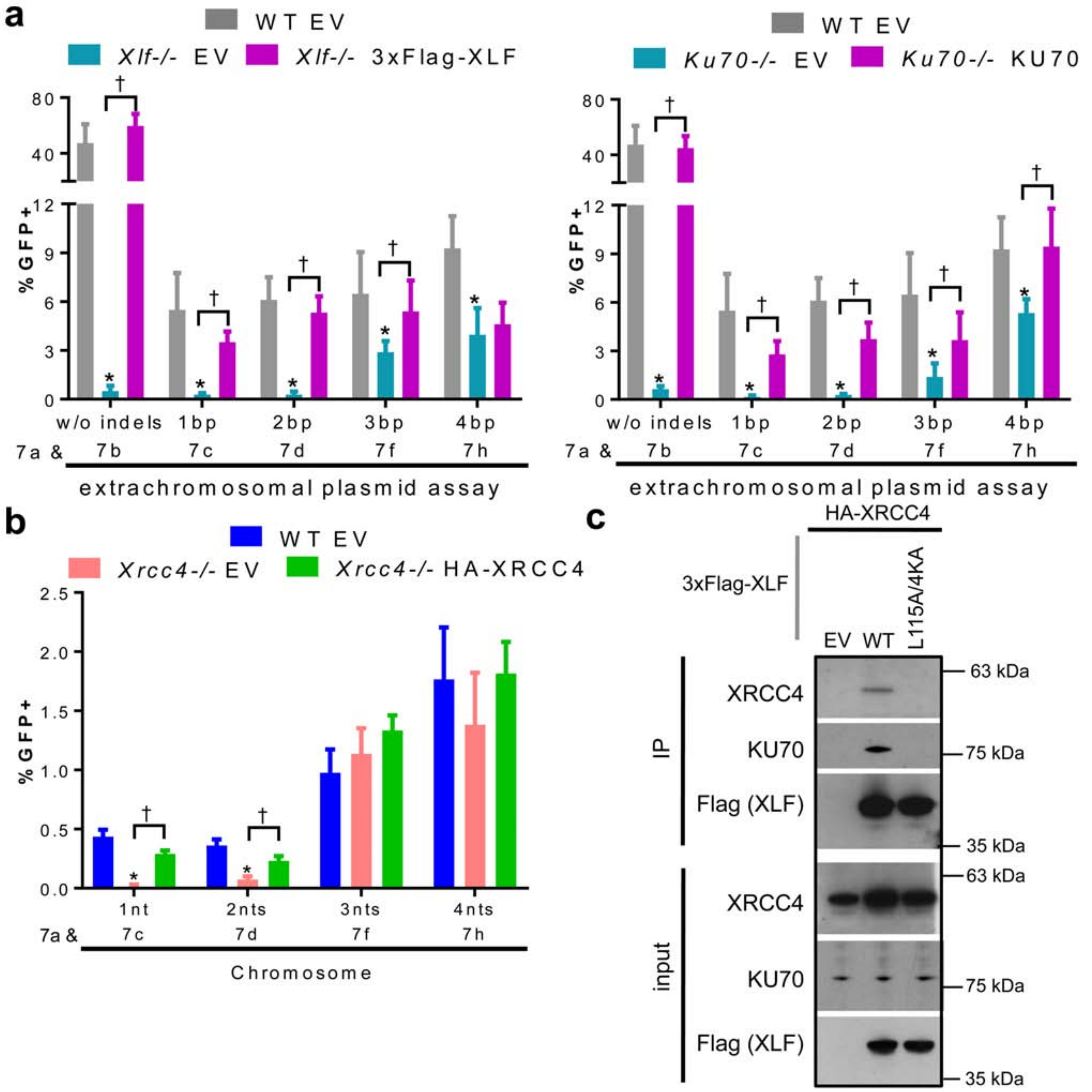

Supplementary Figure 2. Influence of C-NHEJ factors on several EJ assays, and co-IP analysis of XLF WT vs. L115A/4KA. **(a)** Effect of XLF and KU70 on several EJ reporter assays used as extrachromosomal plasmids. The reporters and sgRNAs in Fig. 3a were transfected as plasmids into *Xlf-/-* or *Ku70-/-* and compared to parallel transfections in WT mESCs. Control EV or complementation vector were included. Shown are GFP+ frequencies from these experiments, normalized to transfection efficiency. N=6, error bars indicate s.d. *p≤0.008, WT vs. *Xlf-/-* and *Ku70-/-* using an Unpaired *t*-test, with the Holm-Sidak correction. †p<0.02, control EV vs. complementation vector, using an Unpaired, two-tailed *t*-test. **(b)** Reporters shown in Fig. 3a were integrated into the *Pim1* locus of *Xrcc4-/-* mESCs and transfected with the respective sgRNA/Cas9 plasmids in the presence of a control EV or complementation vector. Shown are the %GFP+ cells normalized to transfection efficiency as in Fig. 1b. %GFP+ values for WT mESCs are the same as in Fig. 3b. N=6. Error bars indicate s.d. *p<0.0001 *Xrcc4-/-* vs. WT using an Unpaired *t*-test with the Holm-Sidak correction. †p<0.0001, control EV vs. the complementation vector, using an Unpaired, two-tailed *t*-test. **(c)** Co-IP analysis of XLF WT and L115A/4KA. Cells were transfected with 3xFlag-XLF (WT, L115A/4KA, or EV) and HA-XRCC4 expression vectors (1200 ng HA-XRCC4 vector per plate, 3 plates each), followed by Flag immunoprecipitation and immunoblotting analysis, as described in the Methods.

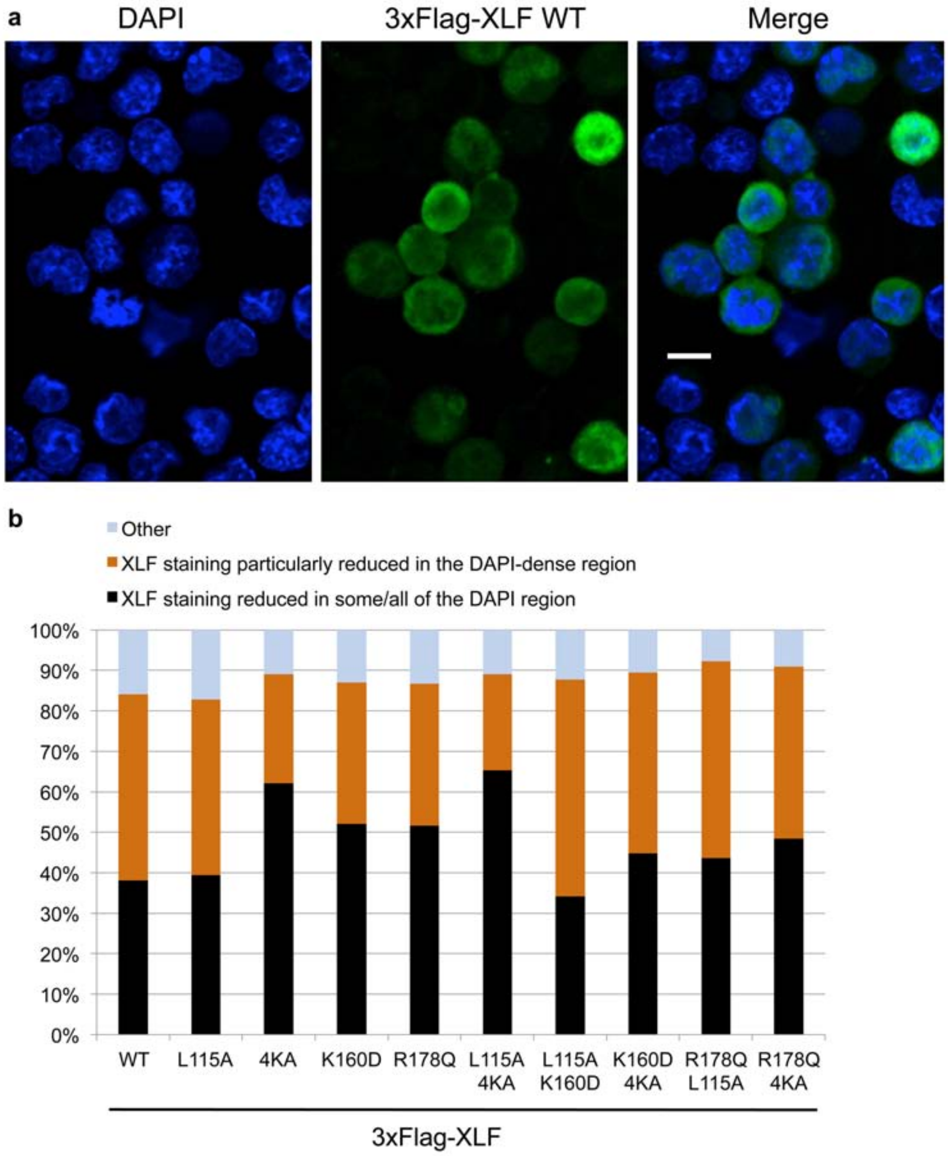

Supplementary Figure 3. XLF staining is often reduced in areas of high DAPI staining, and the staining of the XLF mutants is not obviously different from WT. **(a)** Shown are Flag immunostaining signals from cells transfected with 3xFlag-XLF WT expression vector, with DAPI counterstain. Scale bar = 10 μm. **(b)** Shown are categories of XLF staining patterns from XLF WT and various mutants. N>30 cells each.

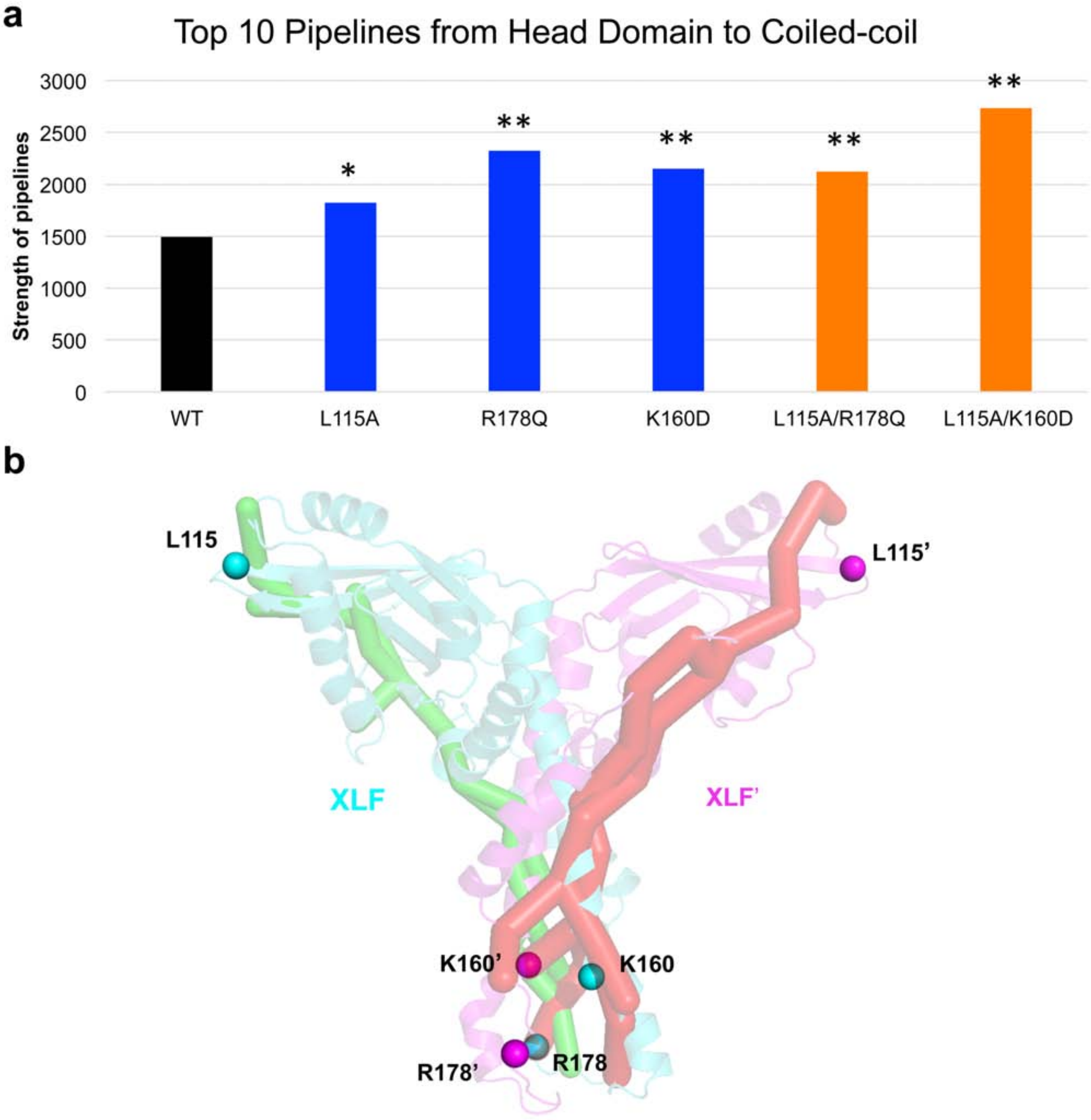

Supplementary Figure 4. Allosteric pipeline analysis. (a) Plot shows the cumulative strength of the top 10 allosteric pipelines that connect the globular head domain to the coiled-coil region for each system. Calculations were performed using custom MATLAB scripts on ensemble trajectories totaling 920 ns for each molecular system. Wilcoxon Rank Sum Test was used to determine significance of differences in cumulative pipeline strength observed between WT and mutant dimers, $^{*}P<0.05$, $^{**}P<0.01$. (b) Mutual Information pipelines from the globular head domain extending to the coiled-coil region are shown for the top (red) and second most-populated (green) pipelines of allosteric communication in the K160D mutant dimer. Each sphere is the alpha carbon of the labeled residue. The monomers are labeled as XLF (cyan) and XLF' (magenta).

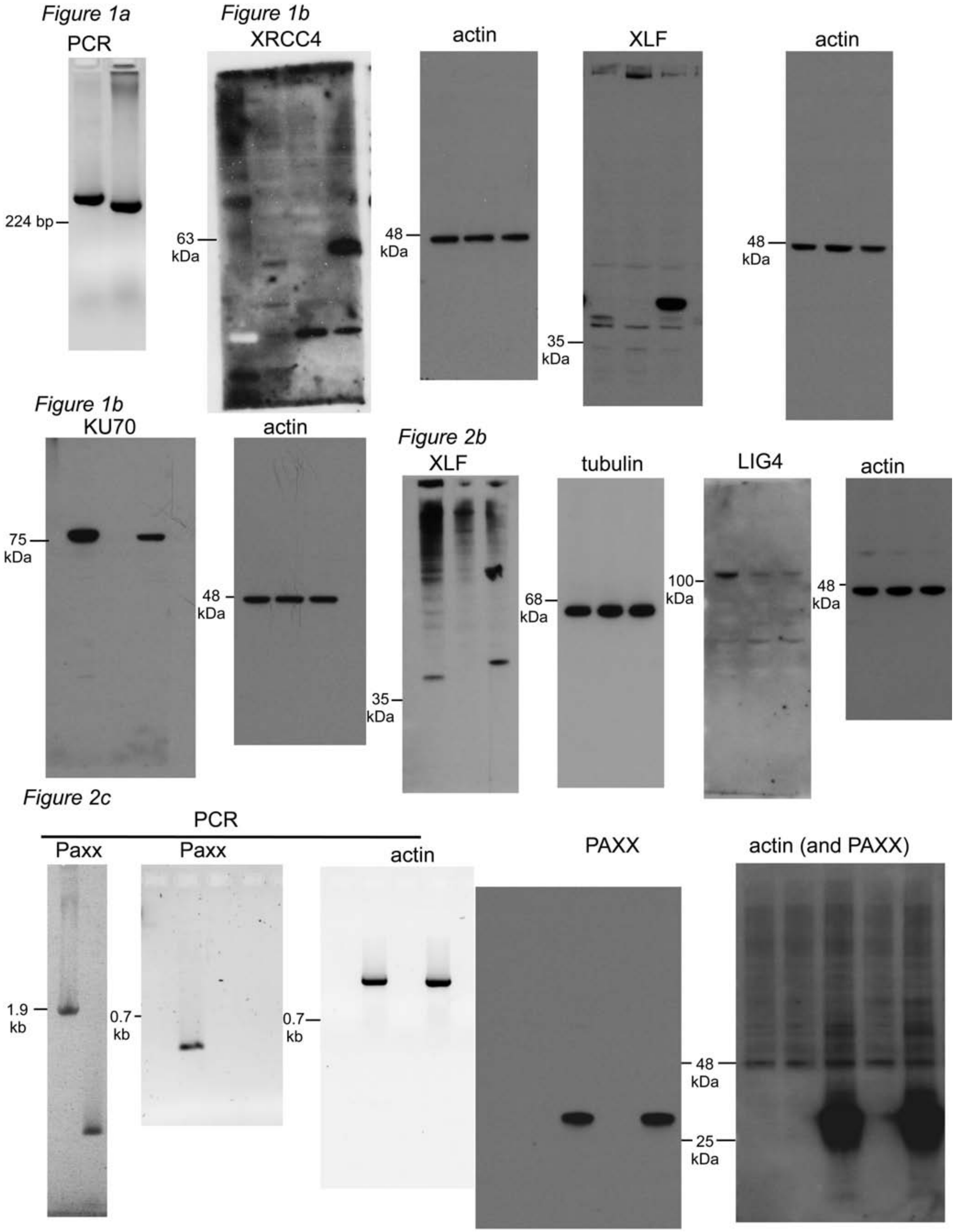

Supplementary Figure 5. Full gels/blots. Full gels/blots from Fig. 1 and Fig. 2 are shown.

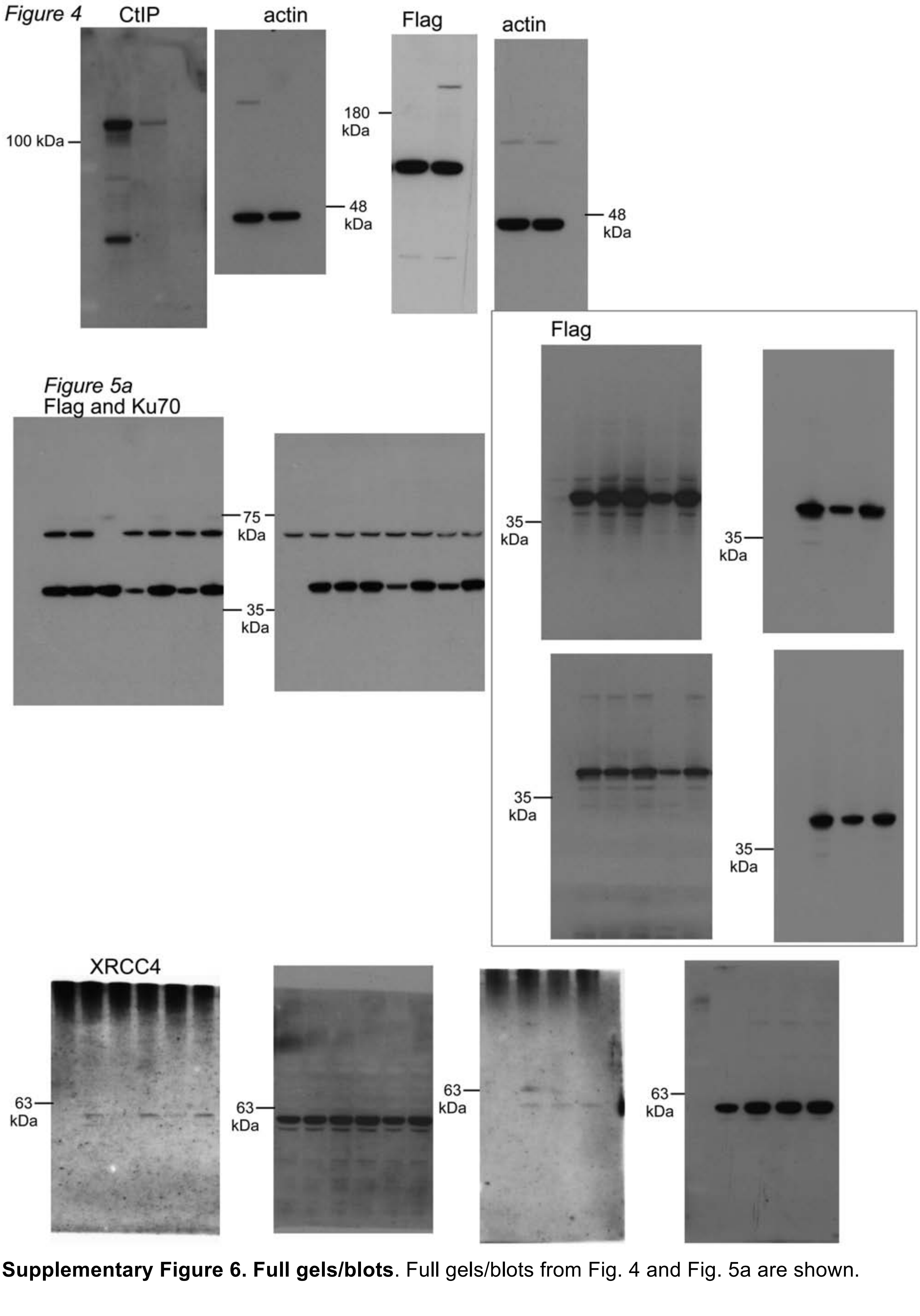

Supplementary Figure 6. Full gels/blots. Full gels/blots from Fig. 4 and Fig. 5a are shown.

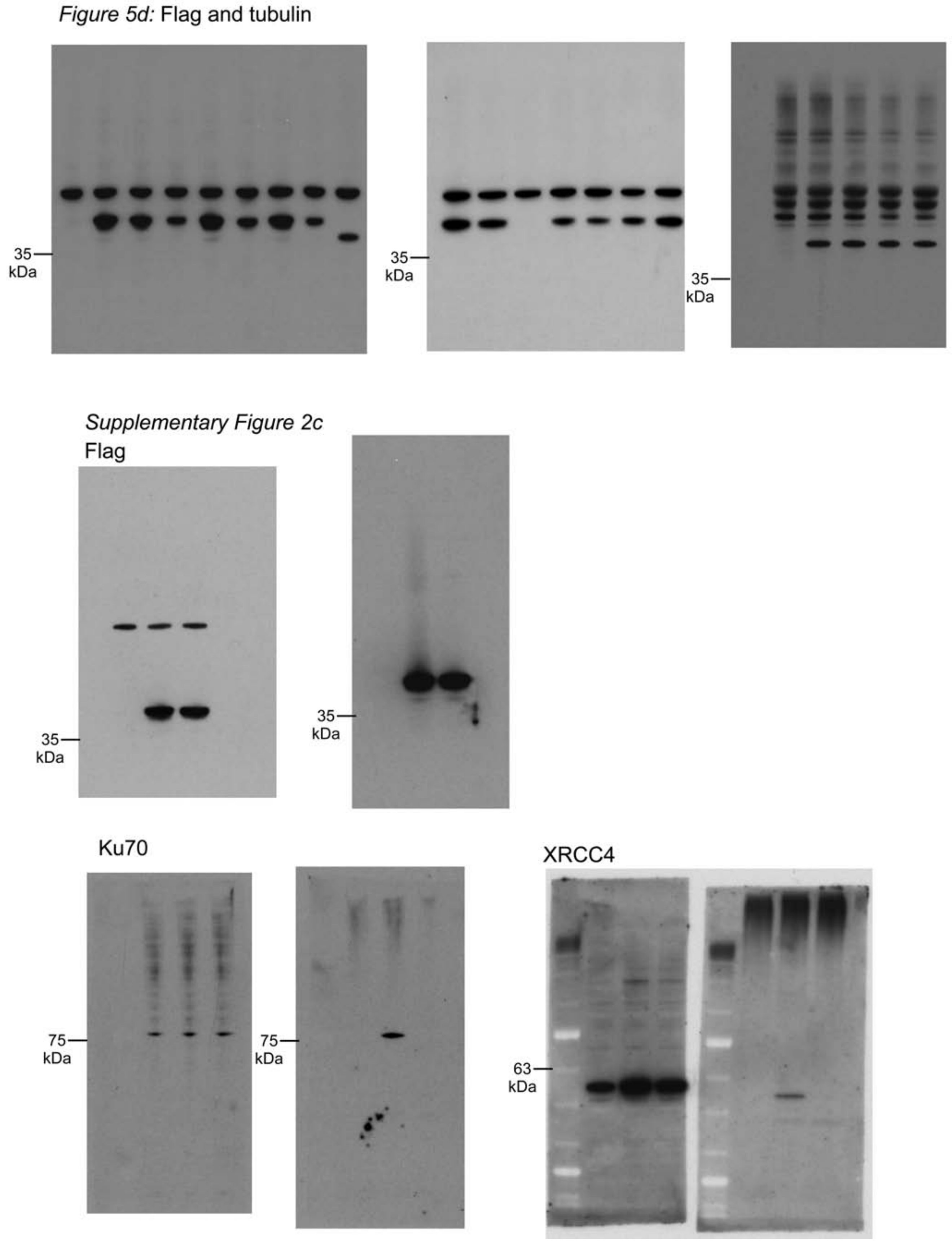

Supplementary Figure 7. Full gels/blots. Full gels/blots from Fig. 5d and Supplementary Fig. 2 are shown.

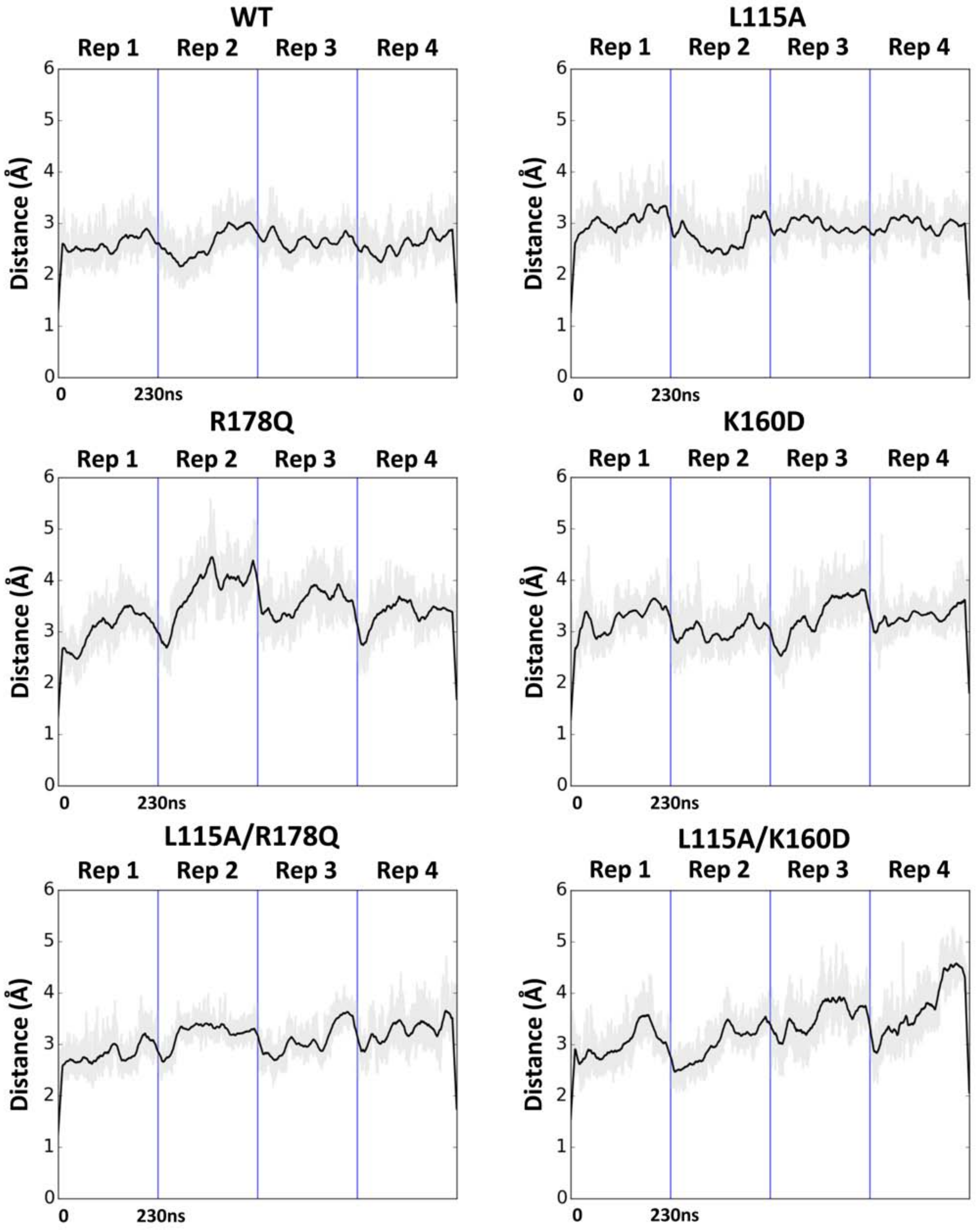

Supplementary Figure 8. Root mean square deviation (RMSD) of Individual Replicates. Analysis of the RMSD of backbone atoms in our MD production runs using the last frame output from equilibration as our reference structure. Each replicate conations 230 ns of data, for a total ensemble of 920 ns per molecular system. Replicates are separated by vertical blue lines.

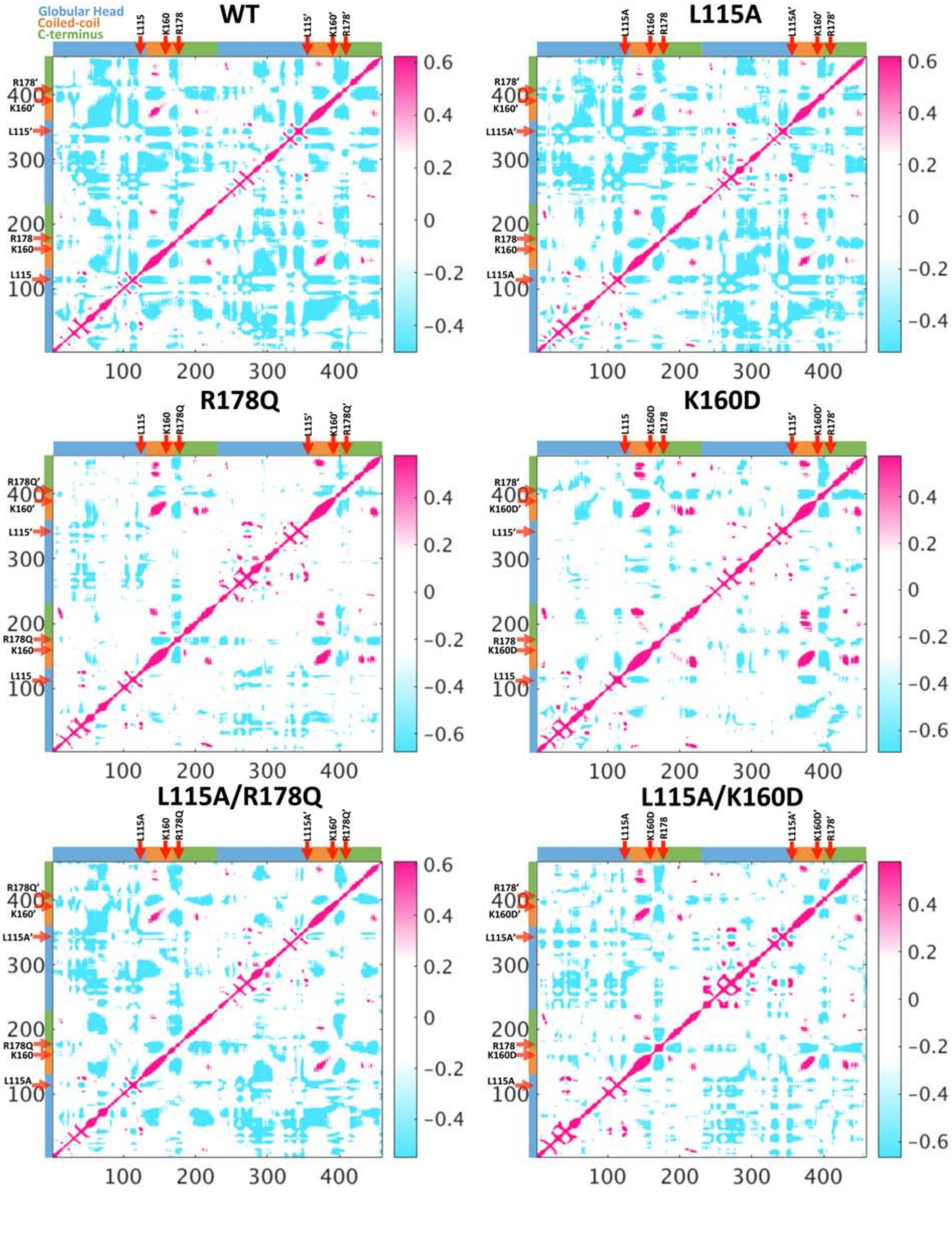

Supplementary Figure 9. Residue-wise Cross Correlation Analysis was performed on each molecular system simulated. The motions of C[alpha] atoms of each residue were compared to those of each other Cα atom in the protein for the entire 920 ns ensemble, composed of four replicate trajectories. The DCCM plot indicates regions of high correlation in magenta, and low correlation in cyan. On the top and left border of each DCCM plot is a guide that shows the structural region corresponding to the residues of the XLF dimer at that position: Globular head domain (blue), Coiled-coil region (orange), and C-terminus (green). Arrows indicate the position of mutated positions L115, K160, and R178. This analysis indicates little variation in the highly correlated positions of the dimer, across the different mutant systems, as compared to WT.

Model	Measurement	Mean	95% one-sided t-test stat
WT	Coiled-Coil Separation	7.5675	0.771014014
	Y167-Y167' Distance	5.8475	0.83246905
	Water Infiltration of Coiled-Coil	2.7025	1.380149918
L115A	Coiled-Coil Separation	7.55	0.807358395
	Y167-Y167' Distance	5.85	0.607004363
	Water Infiltration of Coiled-Coil	2.8025	1.567931729
R178Q	Coiled-Coil Separation	9.3875	1.400235722
	Y167-Y167' Distance	7.58	1.529047053
	Water Infiltration of Coiled-Coil	4.54	1.761069836
K160D	Coiled-Coil Separation	14.24	1.439626764
	Y167-Y167' Distance	12.1525	1.933883665
	Water Infiltration of Coiled-Coil	6.025	2.046432097
L115A/R178Q	Coiled-Coil Separation	7.515	0.764766151
	Y167-Y167' Distance	5.5	0.601262552
	Water Infiltration of Coiled-Coil	2.7675	1.626155003
L115A/K160D	Coiled-Coil Separation	9.16	0.593520403
	Y167-Y167' Distance	7.8525	1.715640833
	Water Infiltration of Coiled-Coil	3.265	1.743252191

Supplementary Table 1. Statistics for Coiled-coil Measurements. Mean and 95% confidence level t-statistic given for Coiled-coil measurements for each dimeric system. Means and t-statistics are calculated from 4 replicates of 230 ns simulation time for each molecular system.
